# Pulmonary Valve Replacement in Adults and Adolescents With Congenital Heart Disease: A United Kingdom and Ireland Survey

**DOI:** 10.1093/icvts/ivaf214

**Published:** 2025-09-24

**Authors:** Chris J Bond, Timothy J Jones, J Andreas Hoschtitzky, Nicola Viola, Mark H D Danton, Nigel E Drury

**Affiliations:** Department of Cardiac Surgery, Queen Elizabeth Hospital Birmingham, Birmingham, United Kingdom; Department of Cardiac Surgery, Queen Elizabeth Hospital Birmingham, Birmingham, United Kingdom; Department of Paediatric Cardiac Surgery, Birmingham Children’s Hospital, Birmingham, United Kingdom; Department of Cardiovascular Sciences, School of Medical Sciences, College of Medicine and Health, University of Birmingham, Birmingham, United Kingdom; Department of Congenital Cardiac Surgery, Royal Brompton Hospital and Evelina London Children’s Hospital, London, United Kingdom; Department of Congenital Cardiac Surgery, Southampton Children’s Hospital, Southampton, United Kingdom; Department of Congenital Cardiac Surgery, Royal Hospital for Children, Glasgow, United Kingdom; Department of Paediatric Cardiac Surgery, Birmingham Children’s Hospital, Birmingham, United Kingdom; Department of Cardiovascular Sciences, School of Medical Sciences, College of Medicine and Health, University of Birmingham, Birmingham, United Kingdom

**Keywords:** congenital heart disease, pulmonary valve replacement, bioprosthesis, pericardial valve, xenograft valve, cardiac surgery

## Abstract

**Objectives:**

Many models of bioprosthesis are available for pulmonary valve replacement in adults with congenital heart disease, but there is a lack of randomized evidence to guide practice. We surveyed congenital cardiac surgeons to establish current practice and willingness to change within a clinical trial.

**Methods:**

An online survey was sent to all consultant congenital cardiac surgeons in adult congenital centres in the United Kingdom and Ireland. Information was sought on preferred prostheses, factors influencing decision-making, implant technique, postoperative anticoagulation, practice variations in adolescents, and willingness to randomize patients to different prostheses within a trial.

**Results:**

Responses were obtained from 27 (69%) surgeons. A total of 19 (70%) preferred an Edwards bovine pericardial valve, most commonly the Inspiris Resilia (7, 26%). Only 2 (7%) favoured the Hancock II valve; the remaining 6 (22%) preferred pulmonary homografts. Data regarding long-term freedom from reintervention (23, 85%) was the most important factor influencing prosthesis choice. A total of 22 (81%) surgeons were willing to randomize adult patients to either a bovine pericardial valve or a porcine xenograft in a clinical trial, with Perimount Magna Ease and Hancock II being the most acceptable, respectively. Willingness to randomize dropped to 11 (41%) surgeons for adolescent patients.

**Conclusions:**

This survey demonstrates heterogeneity in the choice of pulmonary valve prosthesis. Combined with a lack of evidence from clinical trials, our findings support the presence of clinical equipoise. Most surgeons are willing to change practice, suggesting that a pragmatic, multicentre, randomized controlled trial comparing bovine pericardial versus porcine xenograft for pulmonary valve replacement in adults is feasible.

## Graphical abstract

**Figure:**
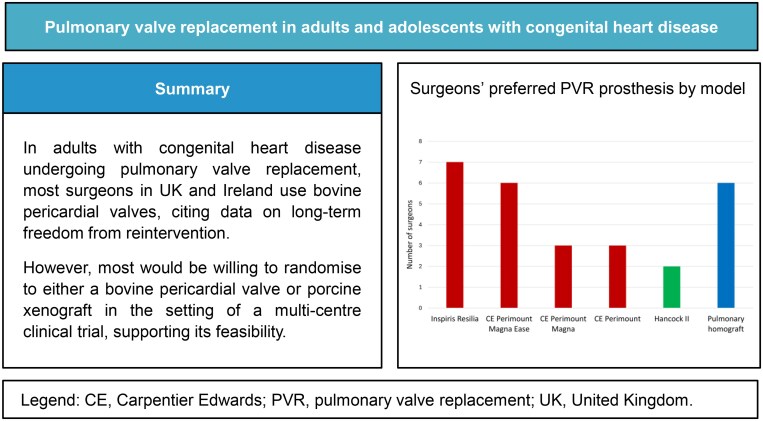

## INTRODUCTION

Pulmonary valve replacement (PVR) is the most common operation in adults with congenital heart disease (CHD) following cardiac surgery in childhood,^1^ and has been shown to improve survival and freedom from ventricular arrhythmias.^2^ However, these patients live with the burden of reintervention, often requiring multiple procedures over a lifetime to treat failing prostheses, which may contribute to late mortality and morbidity, including increased levels of anxiety and depression.^3^ In the recent James Lind Alliance Priority Setting Partnership in CHD, reducing the frequency or need for reoperations in adults with CHD was identified as a key national priority for research.^4^

No bespoke surgical prosthesis is available for the pulmonary position; rather, surgeons implant prostheses designed for the aortic position. Many valve models are available on the market, and most are either bovine pericardial or porcine xenograft valves, with the former used twice as commonly as the latter in the aortic position in England and Wales.^5^ Data on the outcomes of bioprostheses in the pulmonary position are sparse; differences between pulmonary and systemic haemodynamics and mechanisms of structural valve degeneration (SVD) between bovine and porcine prostheses affect the transferability of aortic outcome data to the pulmonary position. Whilst some retrospective observational data suggest differences between valve types in late SVD and freedom from reintervention,^6–10^ there is no clinical trial evidence to support one valve over another. We therefore conducted a survey of congenital cardiac surgeons in the United Kingdom and Ireland to determine current practice and inform the design of a future clinical trial.

## METHODS

As a cross-sectional survey of healthcare providers about their professional clinical practice, in accordance with UK Health Research Authority guidance, formal research ethics committee review was not required. The survey design was informed by our previous experience^11^ and was pre-tested amongst the co-authors, all consultant surgeons, to improve precision and readability. A survey link was sent via email to all 39 consultant congenital cardiac surgeons at each of the 13 level 1 adult CHD centres in the United Kingdom and the Republic of Ireland in September 2023. Non-responders received a second personalized follow-up email after three weeks to prompt completion, and the survey remained open for 5 weeks. Respondents were required to provide consent for anonymous reporting of the information provided. Survey data were collected and managed using REDCap electronic data capture tools (see acknowledgements),^12^ and a full list of questions is available as supplementary material.

In brief, respondents were asked about their usual practice in adults: what is their preferred prosthesis to implant in the pulmonary position; whether this has changed during their consultant career and if so how and why; if they regularly use any other prostheses for PVR; whether their preference differs in adolescents (age 12-17 years); what factors are important in making their choice of pulmonary prosthesis; and for comparison, what is their preferred bioprosthesis in the aortic position. On implantation technique, they were asked about their suture technique, sizing, patching of the pulmonary trunk, and reasons for these choices, including if/when their approach would change. On postoperative management, they were asked about their choice and duration of antiplatelet, anticoagulant and statin therapy following PVR.

Finally, they were asked about their willingness to change practice in the context of a multicentre randomized controlled trial, if there were any bioprosthetic valves that they would be unwilling to implant in the pulmonary position in an adult, and if so, to explain their concerns. Specifically, would they be willing to randomize adults and/or adolescents undergoing PVR to either Carpentier-Edwards Perimount Magna Ease (Edwards Lifesciences, Irvine, CA) or Hancock II (Medtronic, Minneapolis, MN) valve prostheses, whether there were any patient groups that they would be unwilling to randomize, and any additional thoughts on taking part in such a multicentre trial.

Discrete data were summarized by counts and percentages. Data analysis was conducted and outputs generated using R version 4.3.1. The first and corresponding authors had full access to all the data in the study and had final responsibility for the decision to submit for publication.

## RESULTS

The survey was completed by 27 (69%) surgeons, including at least 2 responses from 12 of the 13 level 1 adult CHD centres across United Kingdom and Ireland. All respondents answered all questions, and no duplicate responses were included.

### Prosthesis preferences

**Figure 1.**
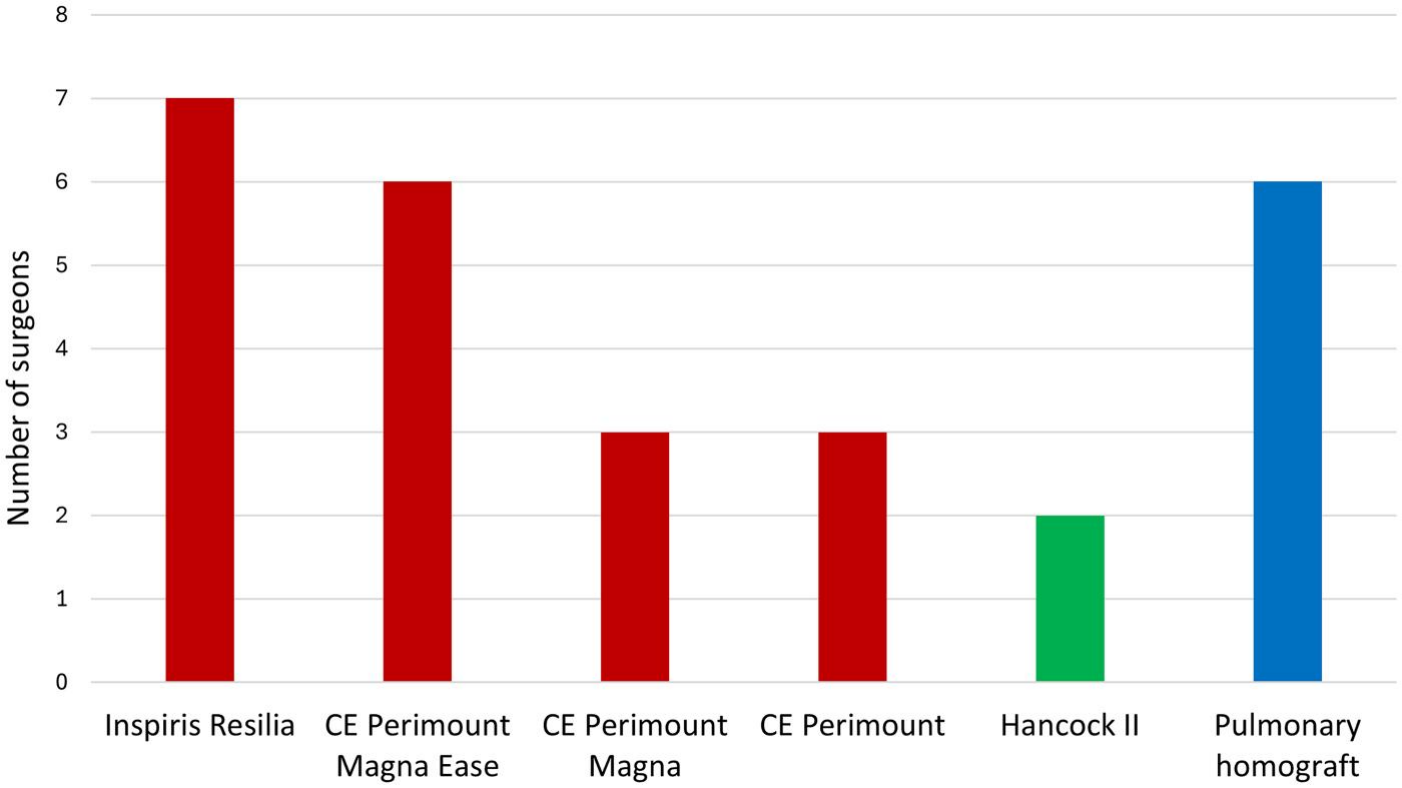
Surgeons’ Preferred Pulmonary Valve Prosthesis in Adults by Model. Red, Bovine Pericardial Valves; Green, Porcine Xenograft; Blue, Homograft. Abbreviation: CE, Carpentier Edwards.

Respondents reported using 6 valve types as their preferred pulmonary prosthesis in adults: 19 (70%) preferred 1 of 4 models of bovine pericardial valve, 6 (22%) used pulmonary homografts, and 2 (7%) opted for the Hancock II porcine xenograft (Figure 1). The most popular model was the Inspiris Resilia (Edwards) bovine pericardial valve, preferred by 7 (26%) surgeons across 5 centres.

Twenty-one (78%) surgeons have changed their preferred prosthesis for PVR during their career. Five (19%) discontinued using pulmonary homografts due to lack of availability, with 1 (4%) having resumed their use due to discontent with outcomes of bovine pericardial valves. A further 5 (19%) stopped using Trifecta (Abbott Laboratories, Chicago, IL) valves due to concern over data showing increased rates of SVD, including in the aortic position.^13^ Two (7%) surgeons switched from a bovine pericardial valve to the Hancock II porcine xenograft due to superior outcomes in studies comparing these valves in the pulmonary position. A total of 21 (78%) surgeons also reported regularly using a model other than their preferred prosthesis in the pulmonary position, with 12 (44%) using a pulmonary homograft and 5 (19%) using the Hancock II valve. Nine (33%) surgeons change their practice in adolescents, with all preferring to use a pulmonary homograft in these patients. The most important factors in making the choice of valve prosthesis for PVR were data on long-term freedom from reintervention (23, 85%) and suitability for future valve-in-valve procedures (21, 78%), as shown in Table 1.

**Table 1.** Factors Important to Surgeons in Making Their Choice of Pulmonary Prosthesis

Factor	Surgeons citing as important (*n* = 27)
Long-term freedom from reintervention	23 (85%)
Suitability for future valve-in-valve implantation	21 (78%)
Ease of implantation	13 (48%)
Long-term survival	9 (33%)
Effective orifice area	9 (33%)
Familiarity	8 (30%)
Prior surgery	6 (22%)
Cost	1 (4%)

In comparison, in the aortic position, 25 (93%) use a bovine pericardial valve, most commonly Inspiris Resilia (14, 52%). No surgeon favours a porcine xenograft in this position, with the remaining 2 (7%) respondents preferring an autograft via the Ross operation. Half of respondents (13, 48%) routinely use the same prosthesis in both pulmonary and aortic positions.

### Surgical technique and perioperative management

Twenty-six (96%) surgeons reported using a single suture implant technique, either continuous (13, 48%), semi-continuous (9, 33%), or both (3, 11%), and 2 (7%) use an interrupted technique. A total of 18 (67%) routinely augment the pulmonary valve annulus using a patch to afford placing a larger diameter prosthesis. Following surgery, 22 (81%) surgeons commence aspirin, 14 (52%) for a fixed duration and 8 (30%) indefinitely, whilst 3 (11%) commence warfarin or a direct oral anticoagulant, and the remaining 2 (7%) do not commence any antiplatelet or anticoagulant therapy. No surgeon reported routinely commencing a statin following PVR.

### Willingness to randomise

Perimount Magna Ease (25, 93%) and Hancock II (23, 85%) were the most acceptable prostheses to which to randomize patients in the setting of a clinical trial (Table 2), with only 1 (4%) respondent unwilling to randomize to either of these 2 valves. When asked specifically, 22 (81%) surgeons would be willing to randomize adults between Perimount Magna Ease and Hancock II; this fell to 11 (41%) for adolescents, with most of those unwilling citing a strong preference for pulmonary homograft in this population. Finally, for 5 (19%) surgeons, there were no currently available valves to which they would be unwilling to randomize.

**Table 2.** Current Valve Prostheses That Surgeons Would be Willing to Implant in the Pulmonary Position in Adults Randomized in a Clinical Trial

Prosthesis model	Manufacturer	Surgeons willing to randomize (*n* = 27)
**Bovine pericardial valve**		
CE Perimount Magna Ease	Edwards	25 (93%)
CE Perimount Magna	Edwards	24 (89%)
Inspiris Resilia	Edwards	23 (85%)
Intuity Elite	Edwards	19 (70%)
Avalus	Medtronic	18 (67%)
Solo Smart	Corcym	13 (48%)
Sorin Mitroflow	Sorin	12 (44%)
**Porcine xenograft**		
Hancock II	Medtronic	23 (85%)
Mosaic	Medtronic	20 (74%)
CE Supra-annular valve	Edwards	20 (74%)
Surgical injectable BioPulmonic	Biointegral	16 (59%)

## DISCUSSION

In this survey of current practice and attitudes towards PVR amongst congenital cardiac surgeons in the United Kingdom and Ireland, we identified that 70% of respondents routinely use one of 4 models of bovine pericardial valve. Data regarding long-term freedom from reintervention and suitability for future valve-in-valve procedures were the most important factors in determining prosthesis selection. Over 80% of surgeons would be willing to randomize adult patients to either a bovine pericardial valve or a porcine xenograft in a multicentre clinical trial, with Perimount Magna Ease and Hancock II identified as the most acceptable valve models, respectively.

To our knowledge, this is the first study to characterize surgeons’ preferences on the selection of valve prosthesis for PVR. In several large series comparing outcomes of valve types in the pulmonary position, there was marked variation in the proportion of patients receiving bovine pericardial valves versus porcine xenografts, ranging from 10% to 93%.^6–10^ Combined, these studies showed 5 models of bovine pericardial valve were utilized in 62% of 2,490 patients across several centres in the United States, South Korea, and Spain centres, alongside 5 models of porcine xenograft. Whilst these studies are subject to potential publication bias, they support our finding that there is clinical equipoise, with no single model dominant and a lack of consensus on which is the best prosthesis for PVR. However, we note that if not for limited availability, 10 surgeons would favour a pulmonary homograft, making this the most popular choice for PVR.

Bioprosthetic valves for use as ventriculo-arterial prostheses are exclusively designed for and tested in the aortic position, with the leading manufacturers conducting no bench testing to replicate conditions in the pulmonary circulation (personal communications: Dr Emmanuel Caillaud and Dr Nacho Martin, Edwards Lifesciences, and Dr Liesbet Boute, Medtronic, March 2024). Implantation in the pulmonary position is not part of the regulatory approvals, and so prostheses are used off-label for PVR worldwide. New valves are introduced into the market with evidence of superior performance in the aortic position, and older models are withdrawn, as occurred recently with the original Carpentier-Edwards Perimount (Edwards), which had good long-term outcomes in the pulmonary position.^8^ This flux disrupts clinical practice, forcing surgeons to change to another model, usually with less late outcome data supporting its use for PVR.

We found the most important factor informing valve choice was long-term freedom from reintervention. This is reflected in some surgeons moving away from the Trifecta valve, which was subsequently withdrawn due to higher rates of SVD.^14^ However, the most commonly preferred prosthesis amongst respondents was the Inspiris Resilia, which has excellent medium-term outcome data in the aortic position,^15^ but there is emerging evidence of an increased incidence of early SVD in the pulmonary position.^10^^,^^16–18^ There is some comparative evidence of superior freedom from reintervention in patients undergoing PVR using a porcine xenograft (Hancock II) compared with a bovine pericardial (CE Perimount) prosthesis (81.3% vs 60.6% at 15 years)^7^ but most patients in this series were children at the time of implantation; younger age has been shown to be strongly related to intervention^8^ so it remains unclear whether this difference is present in adults. These issues underline the risks of adoption of a new prosthesis, the poor transferability of outcome data between the aortic and pulmonary positions, and the need for clinical trial evidence to guide clinical practice and improve outcomes.

Our survey suggests that a trial comparing a bovine pericardial valve with a porcine xenograft in adults with CHD would be widely acceptable to the United Kingdom and Ireland surgical community. Such a trial would need to be pragmatic, considering the variations in practice, surgical factors known to affect rates of SVD, such as valve size and right ventricular outflow tract patching, and suitability for future valve-in-valve reintervention. But it would also need to standardize other aspects of management, such as postoperative antiplatelet/anticoagulation, according to best practice, as variation may also impact the incidence of SVD. Our findings suggest that it should be limited to the adult population, as in adolescents, most surgeons favoured a pulmonary homograft. However, a trial of a bioprosthesis versus pulmonary homograft would be impractical due to the limited availability of the latter, both within a trial and in contemporary clinical practice with increasing demand for reinterventions in a growing adult CHD population.

The strengths of this study include the high response rate and completeness of answers, from surgeons representing almost all adult CHD centres in the United Kingdom and Ireland, to shape the design of a future multicentre clinical trial. Limitations include the applicability of our findings to other healthcare systems and the inability to determine whether the variations in practice described have any impact on patient outcomes.

In conclusion, this survey of practice and attitudes towards PVR in the United Kingdom and Ireland demonstrates heterogeneity in prosthesis selection amongst surgeons. Most favour a bovine pericardial valve, although the most popular model is subject to concerning emerging data on early failure, and there is some observational evidence that porcine xenografts may last longer, especially in younger patients. The current lack of randomized evidence supporting one valve type over another and surgeons’ willingness to change practice within a clinical trial suggests that a pragmatic, multicentre, randomized controlled trial of a bovine pericardial valve versus a porcine xenograft is feasible. This would provide an evidence base to guide valve choice with the potential to improve patient outcomes and reduce the frequency of reinterventions during adulthood, and provide a benchmark against which to compare new valves entering the market.

## Supplementary Material

ivaf214_Supplementary_Data

## Data Availability

The data underlying this article will be shared on reasonable request to the corresponding author.
