# Supplementary material for: Pulmonary Valve Replacement in Adults and Adolescents With Congenital Heart Disease: A United Kingdom and Ireland Survey
Source: Interdiscip Cardiovasc Thorac Surg. 2025 Sep 24;40(10):ivaf214. doi: 10.1093/icvts/ivaf214 (PMC12501497; doi:10.1093/icvts/ivaf214)
Supplement: ivaf214_Supplementary_Data [file ivaf214_supplementary_data.zip › PVR prosthesis survey supplement.pdf]

Survey of Pulmonary Valve Replacement for Congenital Heart Disease in the UK & Ireland

We are conducting this survey on the selection of pulmonary valve prostheses for patients with congenital heart disease in the UK & Ireland to identify variations in practice and willingness to randomise to different prostheses in the context of a potential multi-centre, open label, randomised controlled trial.

Note this work has no funding or involvement from industry.

This survey will take approximately 5-10 minutes to complete. You may Save & Return Later below then follow the link in the email or click 'Returning?' at top right of this page. The survey will close on 27th October 2023.

Your participation is entirely voluntary and you can withdraw at any time by emailing: ChrisBond1@nhs.net.

You can find out what we do with your data in our Privacy Notice.

Please enter your name: __________________________________

At which congenital cardiac surgery centre(s) in the UK or Ireland do you work?

- ◯ Belfast, Royal Victoria Hospital
- ◯ Birmingham, Children's Hospital / Queen Elizabeth Hospital
- ◯ Bristol, Royal Hospital for Children / Bristol Royal Infirmary
- ◯ Dublin, Our Lady's Children's Hospital / Mater Misericordiae Hospital
- ◯ Glasgow, Royal Hospital for Children / Golden Jubilee Hospital
- ◯ Leeds General Infirmary
- ◯ Leicester, Glenfield Hospital
- ◯ Liverpool, Alder Hey Hospital / Liverpool Heart & Chest Hospital
- ◯ London, Evelina London Children's Hospital / St Thomas' Hospital
- ◯ London, Great Ormond Street Hospital / Barts Heart Centre
- ◯ London, Royal Brompton Hospital
- ◯ Newcastle, Freeman Hospital
- ◯ Southampton University Hospital

How many years have you been a consultant?

- ◯ 0-5
- ◯ 6-10
- ◯ 11-15
- ◯ 16-20
- ◯ >20

Please answer the following questions regarding your usual practice.

Which is your preferred prosthesis to implant in the pulmonary position in ADULTS? (select one)

- ◯ Abbott Trifecta aortic valve
- ◯ Abbott Toronto Stentless Porcine Valve (SPV)
- ◯ AutoTissue Matrix P plus valve
- ◯ AutoTissue Matrix P valve
- ◯ BioIntegral Surgical Injectable BioPulmonic valve
- ◯ Carpentier-Edwards Supra-Annular Valve (SAV)
- ◯ Carpentier-Edwards Perimount (models 2700, 2800, 2900)
- ◯ Carpentier-Edwards Perimount Magna (model 3000)
- ◯ Carpentier-Edwards Perimount Magna-Ease (model 3300FTX)
- ◯ Edwards Intuity Elite
- ◯ Edwards Inspiris Resillia
- ◯ Medtronic Avalus
- ◯ Medtronic Hancock II aortic valve
- ◯ Medtronic Mosaic aortic bioprosthesis
- ◯ Sorin Mitroflow
- ◯ Sorin Solo Smart Tissue Med valve
- ◯ Pulmonary homograft
- ◯ Aortic homograft
- ◯ Other

Other - please provide details: __________________________________

Has your preferred pulmonary prosthesis changed during your consultant career?

- ◯ Yes
- ◯ No

How and why did your preference change? __________________________________

Other than your preferred prosthesis selected above, do you regularly use any other prostheses in the pulmonary position?

- ◯ Yes
- ◯ No

Which other prostheses do you regularly use in the pulmonary position? (select all that apply)

- ☐ Abbott Trifecta aortic valve
- ☐ Abbott Toronto Stentless Porcine Valve (SPV)
- ☐ AutoTissue Matrix P plus valve
- ☐ AutoTissue Matrix P valve
- ☐ BioIntegral Surgical Injectable BioPulmonic valve
- ☐ Carpentier-Edwards Supra-Annular Valve (SAV)
- ☐ Carpentier-Edwards Perimount (models 2700, 2800, 2900)
- ☐ Carpentier-Edwards Perimount Magna (model 3000)
- ☐ Carpentier-Edwards Perimount Magna-Ease (model 3300FTX)
- ☐ Edwards Intuity Elite
- ☐ Edwards Inspiris Resillia
- ☐ Medtronic Avalus
- ☐ Medtronic Hancock II aortic valve
- ☐ Medtronic Mosaic aortic bioprosthesis
- ☐ Sorin Mitroflow
- ☐ Sorin Solo Smart Tissue Med valve
- ☐ Pulmonary homograft
- ☐ Aortic homograft
- ☐ Other

Other - please provide details: __________________________________

Does your practice differ in ADOLESCENTS (age 12-17 years)?	◯ Yes<br>◯ No
Please describe how your choice of prosthesis is different in adolescents:	__________________________________
Which factor(s) are important to you in making the choice of pulmonary prosthesis? (select all that apply)	☐ Data on long-term freedom from reintervention<br>☐ Data on long-term survival<br>☐ Effective orifice area for a given valve diameter<br>☐ Ease of implantation<br>☐ Familiarity<br>☐ Previous surgery<br>☐ Suitability for future percutaneous valve-in-valve procedures<br>☐ Cost of prosthesis<br>☐ Other
Other - please provide details:	____________________________
In comparison, which is your preferred bioprosthesis to implant in the aortic position in ADULTS? (select one)	◯ Abbott Trifecta aortic valve<br>◯ Abbott Toronto Stentless Porcine Valve (SPV)<br>◯ AutoTissue Matrix P plus valve<br>◯ AutoTissue Matrix P valve<br>◯ BioIntegral Surgical Injectable BioPulmonic valve<br>◯ Carpentier-Edwards Supra-Annular Valve (SAV)<br>◯ Carpentier-Edwards Perimount (models 2700, 2800, 2900)<br>◯ Carpentier-Edwards Perimount Magna (model 3000)<br>◯ Carpentier-Edwards Perimount Magna-Ease (model 3300FTX)<br>◯ Edwards Intuity Elite<br>◯ Edwards Inspiris Resillia<br>◯ Medtronic Avalus<br>◯ Medtronic Hancock II aortic valve<br>◯ Medtronic Mosaic aortic bioprosthesis<br>◯ Sorin Mitroflow<br>◯ Sorin Solo Smart Tissue Med valve<br>◯ Other
Other - please provide details:	____________________________

Pulmonary valve implantation technique

Which implantation technique(s) do you routinely use for pulmonary valve replacement? (select all that apply)	☐ Continuous single suture<br>☐ Semi-continuous running stitch but requiring more than one suture<br>☐ Simple interrupted<br>☐ Interrupted pledgeted everting, outside to inside annulus<br>☐ Interrupted non-pledgeted everting, outside to inside annulus<br>☐ Interrupted pledgeted inverting, inside to outside annulus<br>☐ Interrupted non-pledgeted inverting, inside to outside annulus

If you routinely use more than one technique, please briefly describe your decision-making process.

____

When performing pulmonary valve replacement in an annulus of normal diameter (i.e. not atretic, hypoplastic or diminutive for the patient's size), do you routinely:

- ◯ Size a prosthesis to the existent annulus
- ◯ Patch the pulmonary trunk to augment the diameter and thereby upsize the prosthesis
- ◯ Use a right ventricular to pulmonary artery conduit e.g. pulmonary or aortic homograft
- ◯ Other

Other - please provide details:

____

Please explain your reasons for this choice of technique and if/when you may change your approach.

____

Postoperative management

Following pulmonary valve replacement, what is your usual strategy regarding anti-platelet and/or anti-thrombotic therapy? (select one)

- ◯ Aspirin (or other antiplatelet) for a fixed duration
- ◯ Aspirin (or other antiplatelet) indefinitely
- ◯ Warfarin (or other Vitamin K antagonist) for a fixed duration
- ◯ Warfarin (or other Vitamin K antagonist) for a fixed duration followed by transition to aspirin (or other antiplatelet)
- ◯ Warfarin (or other Vitamin K antagonist) indefinitely
- ◯ Novel oral anti-coagulant for a fixed duration
- ◯ Novel oral anti-coagulant for a fixed duration followed by transition to aspirin (or other antiplatelet)
- ◯ Novel oral anti-coagulant indefinitely
- ◯ Other

Other - please provide details:

____

How long would this fixed duration usually be?

____

Following pulmonary valve replacement, do you routinely commence a statin if the patient was not previously taking one?

- ◯ Yes
- ◯ No

We would like to assess the willingness of the congenital surgical community to change practice within the context of a multi-centre randomised controlled trial. The proposed trial would be designed and conducted collaboratively, ideally at all interested centres in the UK & Ireland.

Are there any bioprosthetic valves that you be unwilling to implant in the pulmonary position in an adult if they were randomised by prosthesis type in a clinical trial? (select all that apply)

- ☐ Abbott Trifecta aortic valve
- ☐ Abbott Toronto Stentless Porcine Valve (SPV)
- ☐ AutoTissue Matrix P plus valve
- ☐ AutoTissue Matrix P valve
- ☐ BioIntegral Surgical Injectable BioPulmonic valve
- ☐ Carpentier-Edwards Supra-Annular Valve (SAV)
- ☐ Carpentier-Edwards Perimount (models 2700, 2800, 2900)
- ☐ Carpentier-Edwards Perimount Magna (model 3000)
- ☐ Carpentier-Edwards Perimount Magna-Ease (model 3300FTX)
- ☐ Edwards Intuity Elite
- ☐ Edwards Inspiris Resillia
- ☐ Medtronic Avalus
- ☐ Medtronic Hancock II aortic valve
- ☐ Medtronic Mosaic aortic bioprosthesis
- ☐ Sorin Mitroflow
- ☐ Sorin Solo Smart Tissue Med valve
- ☐ Other
- ☐ None - willing to implant any of the above, if available

Other - please provide details:

____

Please briefly explain your concerns about these valve(s).

____

Specifically, would you be willing to randomise ADULTS undergoing pulmonary valve replacement to either Carpentier-Edwards Perimount Magna-Ease or Medtronic Hancock II valve prostheses?

- ◯ Yes
- ◯ No

Please explain your concerns about such a trial.

____

In such a trial, with the following eligibility criteria:

- Inclusion: diagnosis of adult congenital heart disease, aged ≥18 years
- Exclusion: infective endocarditis, cardiac trauma, carcinoid heart disease, emergent or salvage surgery

are there any other patient groups you would NOT be willing to randomise?

____

Would you be willing to randomise ADOLESCENTS (age 12-17 years) undergoing pulmonary valve replacement to either Carpentier-Edwards Perimount Magna-Ease or Medtronic Hancock II valve prostheses?

- ◯ Yes
- ◯ No

Please explain your concerns about such a trial.

____

Finally, we would be most grateful for your thoughts on taking part in a multi-centre randomised controlled trial of prosthetic valve type in the pulmonary position in patients with CHD in the UK & Ireland. ______________________________________
